# Physicochemical and Antimicrobial Evaluation of Bacterial Cellulose Derived from Spent Tea Waste

**DOI:** 10.3390/polym17182521

**Published:** 2025-09-18

**Authors:** Cem Gök, Arzum Işıtan, Massimo Bersani, Paolo Bettotti, Laura Pasquardini, Michele Fedrizzi, Davide D’Angelo, Havva Boyacıoğlu, Ahmet Koluman

**Affiliations:** 1 Department of Biomedical Engineering, Izmir Bakırçay University, 35665 Izmir, Türkiye; 2 Biomedical Technologies Design Application and Research Center, Izmir Bakırçay University, 35665 Izmir, Türkiye; 3 Department of Mechanical Engineering, Pamukkale University, 20160 Denizli, Türkiye; 4 Center for Sensors and Devices, Fondazione Bruno Kessler, 38123 Trento, Italy; bersani@fbk.eu (M.B.); mfed@fbk.eu (M.F.); 5 Bilgece Mühendislik Geri Dönüşüm Ltd., Şti, 20020 Denizli, Türkiye; 6 Nanoscience Laboratory, Department of Physics, University of TrentoPovo, 38123 Trento, Italy; paolo.bettotti@unitn.it; 7 Indivenire srl, 38123 Trento, Italy; l.pasquardini@indiveni.re; 8 Cosvitec Scarl, 80142 Naples, Italy; davidedangelo@cosvitec.eu; 9 Department of Chemical Engineering, Pamukkale University, 20160 Denizli, Türkiye; hboyacioglu@pau.edu.tr; 10 Department of Biomedical Engineering, Pamukkale University, 20160 Denizli, Türkiye; akoluman@pau.edu.tr

**Keywords:** food waste, *Komagataeibacter xylinus*, antimicrobial activity, medical electrode applications, sustainable development goals, circular bioeconomy

## Abstract

Bacterial cellulose (BC) is a high-purity biopolymer with excellent physicochemical and mechanical properties, including high crystallinity, water absorption, biocompatibility, and structural tunability. However, its large-scale production is hindered by high substrate costs and limited sustainability. In this study, spent black tea waste was utilized as a low-cost and eco-friendly carbon source for BC synthesis by *Komagataeibacter xylinus* ATCC 53524 under varying initial pH conditions (4–9). Six different BC membranes were produced and systematically characterized in terms of mechanical strength, water absorption capacity, electrical conductivity, antimicrobial performance, and polyvinyl alcohol (PVA) attachment efficiency. Morphological and chemical analyses were conducted using SEM and FTIR techniques to investigate pH-induced structural variations. The results revealed that the BC6 sample (pH 6) exhibited the highest tensile strength (2.4 MPa), elongation (13%), PVA incorporation (12%), and electrical conductivity, confirming the positive impact of near-neutral conditions on nanofiber assembly and functional integration. In contrast, the BC4 sample (pH 4) demonstrated strong antimicrobial activity (log reduction = 3.5) against *E. coli*, suggesting that acidic pH conditions enhance bioactivity. SEM images confirmed the most cohesive and uniform fiber morphology at pH 6, while FTIR spectra indicated the preservation of characteristic cellulose functional groups across all samples. Overall, this study presents a sustainable and efficient strategy for BC production using food waste and demonstrates that synthesis pH is a key parameter in tuning its functional performance. The optimized BC membranes show potential for biomedical, flexible electronic, and antibacterial material applications, particularly in wearable electrode technologies.

## 1. Introduction

Both agricultural waste and food wastage are significant global challenges today. These two issues pose serious environmental and economic challenges that hinder the achievement of sustainable development goals. Food waste not only threatens food security but also causes great damage to the environment worldwide. However, agricultural waste, when recycled and utilized, can provide environmental benefits and form key components of sustainable practices [1,2,3,4].

Food waste refers to the reduction in the quantity or quality of food due to decisions and actions by retailers, food service providers, and consumers. It includes food and inedible parts removed from the supply chain for recovery or disposal through methods such as composting, anaerobic digestion, bioenergy production, incineration, or landfilling [5]. In the EU, over 59 million tons of food waste (132 kg per inhabitant) are generated annually, valued at 132 billion euros, while more than 42 million people struggle to afford a quality meal every second day [6,7]. Globally, nearly one-third of food produced for human consumption is lost or wasted, with 14% lost during the post-harvest to retail stages [5]. Food waste intensifies the environmental impact of production and consumption. The European Commission’s Circular Economy Action Plan promotes resource efficiency and sustainability, with its 2023 revision adding indicators on material footprint and resource use [8]. The main objective is to transform low-value residues and waste into valuable products [9]. By following circular economy principles and better utilizing or reusing existing resources, the shift from a fossil-based to a bio-based economy accelerates. Biorefineries, utilizing diverse biomass feedstocks, employ various technologies to produce energy, biofuels, chemicals, and materials [10].

Cellulose, a widely abundant biopolymer derived from plants, algae, bacteria, and tunicates, provides structural integrity to plant cell walls. Its biodegradability and renewability make it vital for sustainable development, reducing reliance on fossil fuels. Structural diversity influences its properties and applications across various fields [11,12]. Classified by source and structure, common forms include microcrystalline cellulose (MCC), nanocrystalline cellulose (NCC), and bacterial cellulose (BC) [11,12]. BC is valued for its high tensile strength, water retention, and biocompatibility, making it ideal for medical applications [13]. BC-based soft and moisturizing film electrodes are designed for biopotential recording. It is also reported that the water in BC can continuously moisturize the skin, reduce contact impedance, and in addition, compared to gel electrode patches, BC electrodes do not cause skin discomfort when worn for long periods of time [14].

As a sustainable alternative to traditional cellulose sources, BC production using organic waste aligns with circular economy principles [15,16]. First described in 1886 by Adrian J. Brown, BC is synthesized by various bacteria like *Rhizobium leguminosarum*, *Burkholderia* spp., *Escherichia coli* (*E. coli*), and *Salmonella enterica*, with *Komagataeibacter xylinus* (*K. xylinus*) being the most studied due to its high yield and ability to utilize diverse carbon sources [17]. *K. xylinus* belongs to the acetic acid bacteria (AAB) group within the *Acetobacteraceae* family and is commonly found in fermented foods, fruits, and vegetables [17,18]. Its biosynthesis follows the pentose phosphate pathway and Krebs cycle, with acid tolerance optimizing production. Traditional vinegars serve as natural AAB sources, where *K. xylinus* can be identified via biochemical and molecular analyses such as 16S rRNA sequencing.

Tea (*Camellia sinensis*) is the second most consumed beverage globally, involving over 13 million people in its industry. Black and green tea dominate production (98%), with major producers including China, India, Kenya, Sri Lanka, and Türkiye. Black tea accounts for 60% of production, followed by green tea at 30%. In 2013, global tea production reached 5.07 million tons, with black and green tea outputs rising by over 5%. Black tea production, which was previously projected to grow by 2.9% annually to 4.17 million tons by 2023 alongside a 3% annual increase in consumption to 4.14 million tons, is now expected to expand at a slower pace than in the past decade, rising by 1.6% per year to reach 4.42 million tons by 2032 [19,20,21,22].

**Figure 1:**
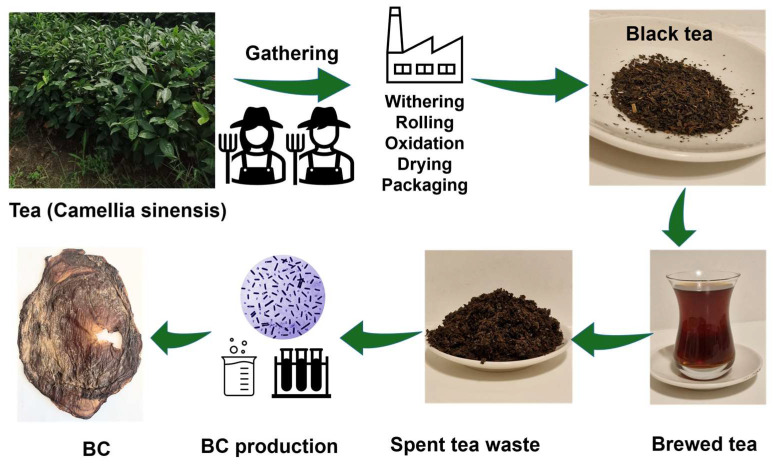
An illustration of BC production from waste generated during the production and processing of tea.

As illustrated in Figure 1, tea waste is generated throughout the tea production, including withering, crushing, fermentation, drying, and packaging, comprising fiber, protein, caffeine, and polyphenols. About 90% of tea leaves become waste post-processing, posing environmental and economic challenges. India produces 190,000 tons of tea waste annually, while Türkiye’s Black Sea region generates 30,000 tons [20,23,24]. It consists of fiber, protein, caffeine, polyphenols, and other bioactive compounds. If not properly managed, tea waste can pollute water, soil, and air. To address these issues, tea waste can be valorized through extraction methods to recover bioactive compounds like polyphenols. It has potential applications in bioenergy production, adsorbents, food additives, and functional foods [20,23]. “Spent tea leaves” (STLs) from brewed tea also contribute to waste generation, requiring disposal efforts and economic resources [23]. Sustainability initiatives, including the “waste to wealth” concept, aim to convert tea waste into valuable products, reducing environmental impact and supporting circular economy practices. Additionally, black tea leaf silage is rich in fiber, while green tea leaf silage has high phenolic and lactic acid content, making tea waste an important source of nutrients and bioactive compounds [20]. BC extraction from fermented black tea is considered to be economical compared to conventional media as it naturally contains many essential nutrients and complementary components such as ethanol and vitamins. This is expected to make production cheaper compared to the process using ethanol-supplemented conventional media [13]. There are studies in which *K. xylinus* was isolated from fermented tea (kombucha) and used in BC production [16,25,26]. In these studies under various culture conditions, focusing on carbon sources, temperature, and pH are also reported to be very influential on BC structure and properties.

The United Nations and the European Union are working hard to address the issues through various policies and programs such as the Sustainable Development Goals (SDGs) [27], the European Green Deal [28], and the Circular Economy Plan [29]. These ambitious plans require investments that will reduce emissions, promote the use of renewable energy sources and environmentally friendly technologies, and support industry in its innovation while implementing the green transition across all sectors. In this context, the Food Chase Project [30], supported by the European Union, is working on the creation of “Food Supply Chain Ecosystems for Sustainability” and reducing food loss and waste throughout the supply chain. The project brings together higher education and vocational education and training, research, the public sector and the business sector to design and build new, innovative and multidisciplinary approaches to food supply chain training and development of new skills based on market needs, from farm to post-use.

In order to contribute to the aforementioned aims and objectives, within the scope of the FOOD CHASE project, this study investigated the usability of BCs from spent black tea in medical electrode applications. Although there are studies in the literature on BC production of *K. xylinus* bacteria with tea, there are few studies on BC production with this bacterium from black tea as an electrode material, whose waste amount reaches millions of tons [13,31]. In this study, six different BCs were obtained from black tea wastes by selecting different initial pH levels and thickness measurement, strength, conductivity, water absorption capacity, colorfastness, antimicrobial properties, and PVA attachment tests were carried out to examine their usability as electrode materials.

## 2. Materials and Methods

### 2.1. Production of BC from Spent Black Tea and ITS Purification

Neither the bacterial strain nor the tea waste underwent pre-treatment. All chemicals and reagents used in the process were of laboratory grade and used without further purification. The waste tea was obtained from the tea kiosk operating within the Faculty of Technology at Pamukkale University.

*K. xylinus* (ATCC 53524) was obtained from the American Type Culture Collection (ATCC) and maintained on agar slants at 4 °C. For cultivation, the strains were inoculated in Hestrin-Schramm (HS) medium containing 2% glucose, 0.5% yeast extract, 0.5% peptone, 0.27% disodium phosphate, and 0.11% citric acid, adjusted to pH 6.0 [32]. The used tea leaves (brewed/spent) were washed with sterilized water. Next, spent black tea (5 g) was added to this solution. The solution was left to stand at room temperature to enable the components of the black tea to infuse properly into the water. Tea leaves were removed by filtration with sterile sieve after 15 min. BC membranes were produced by inoculating 1% (*v*/*v*) of the activated bacterial strain into 100 mL of HS medium in sterile 250 mL Erlenmeyer flasks (Isolab, Eschau, Germany). The culture was incubated varying initial pH values (4.0–9.0) by adding acetate or citrate statically at 30 °C for 15 days. The samples were numbered BC4, BC5, BC6, BC7, BC8, and BC9 depending on the initial pH values. After cultivation, the BC membrane was harvested and purified by washing with deionized water to remove residual medium components and bacterial cells. The membrane was then treated with 0.1 M NaOH at 80 °C for 2 h to remove any remaining impurities, followed by thorough rinsing with deionized water until neutral pH was achieved. The purified BC membranes were air-dried at room temperature for 24 h and stored in a desiccator until further analysis.

### 2.2. Thickness Measurement

The thickness of the BC membranes was measured using a digital micrometer (Mitutoyo, Kawasaki, Japan) with an accuracy of ±0.001 mm. Thickness measurements were performed after incubating the culture at 30 °C for 15 days at different initial pH values and subsequent purification and drying steps. Measurements were taken at five random locations on each sample, and the thickness is reported as mean average and standard deviation.

### 2.3. Tensile Strength and Elongation

Tensile strength and elongation at break were determined according to ASTM D-882-97 using a universal testing machine (Instron 3366, Instron Co., Norwood, MA, USA) equipped with a 500 N load cell [33]. Samples were maintained at ambient temperature in a laminar cabinet for at least 2 weeks to reach equilibrium crystallization. Rectangular samples (10 mm × 50 mm) were cut from the BC membrane and clamped between the grips of the testing machine with an initial grip separation of 20 mm. The samples were stretched at a constant crosshead speed of 5 mm/min until fracture. Tensile strength (MPa) was calculated as the maximum load divided by the cross-sectional area of the sample, and elongation (%) was determined as the percentage increase in length at the point of fracture relative to the original length. Measurements were conducted at ambient temperature. Three separate measurements were made and averaged.

### 2.4. Colorfastness Tests

As a stage of the usability of BCs as electrode material, following ASTM-recommended procedures, the dyed samples were evaluated for various fastness properties. The specific test methods applied included colorfastness to washing/laundering (AATCC 61-2013) [34], colorfastness to rubbing (ISO 105-X12:2016) [35], and colorfastness to perspiration (AATCC TM 15-2021) [36].

Washing fastness was evaluated using a Launder-Ometer (SDL Atlas, LLC, Rock Hill, SC, USA) according to AATCC Test Method 61-2013. The samples were subjected to 10 washing cycles at 40 °C, and the color change was assessed on a gray scale of 1 to 5, where 1 indicates significant color loss and 5 indicates no color change.

Rubbing fastness was assessed using a crock meter (SDL Atlas, LLC, Rock Hill, SC, USA) according to ISO 105-X12:2016 in dry procession for testing the transference of color from the surface of one material to another. The number of rubbing cycles required to cause visible wear on the BC membrane surface was recorded.

The colorfastness to perspiration test was performed according to the AATCC test method 15-2021 to examine how much its color changes when in contact with body perspiration. Test specimens were cut to dimensions of 60 × 60 ± 2 mm. The gray scale was utilized to assess the samples’ fastness performance in this test. The gray scale comprises nine possible ratings: 5, 4–5, 4, 3–4, 3, 2–3, 2, 1–2, and 1, where a rating of 5 indicates “Excellent” fastness, while a rating of 1 signifies “Very Poor” fastness.

### 2.5. Electrical Conductivity Measurement

The electrical conductivity of the BC membrane was measured using a four-point probe conductivity meter (Keithley 2450, Keithley Instruments, Cleveland, OH, USA). The samples were cut into 10 × 10 mm^2^ and placed between the probes. A constant current was applied, and the voltage drop across the sample was recorded. The conductivity (S/m) was calculated using Ohm’s law and the sample dimensions. Measurements were conducted at room temperature, and the conductivity was calculated using Equation (1) [37]:

(1) $$\sigma =\frac{1}{R.A/L}$$

where *σ* is the conductivity (S/cm), *R* is the resistance (Ω), *A* is the cross-sectional area (cm^2^), and *L* is the sample thickness (cm).

### 2.6. Water Absorption Capacity

BC is known for its exceptional water absorption and retention capacity compared to its plant-based counterpart [13]. The water absorption properties are closely linked to the material’s porosity, crystallinity, and surface area. BC samples maintained at ambient conditions in a laminar chamber for 2 weeks were cut into small pieces (4 × 4 cm) and weighed. The water absorption capacity of the BC membrane was determined by soaking the samples in deionized water at room temperature for 24 h. The samples were then removed, gently blotted with filter paper to remove excess surface water and weighed. The water absorption capacity (%) was calculated using Equation (2):

(2) $$Water\;absorbtion\;capacity\left(\%\right)=\frac{W_{w}-W_{d}}{W_{d}}\times 100$$

where *W_w_* is the weight of the wet sample and *W_d_* is the weight of the dry sample.

### 2.7. Antimicrobial Activity

The antibacterial activity of the specimens was evaluated using the dynamic contact method based on the ASTM E2149-13a standard [38]. This method allows for the quantitative assessment of the reduction in bacterial concentration when in contact with an antimicrobial surface under shaking conditions. *Escherichia coli* (*E. coli*) (ATCC 25922), a Gram-negative bacterium, was used as the test organism. A bacterial suspension was prepared in 0.9% NaCl solution and adjusted to an initial concentration of approximately 8 log CFU/mL (10^8^ CFU/mL). Square specimens measuring 20 × 20 mm were placed in sterile containers containing 100 µL of the bacterial suspension. The samples were incubated for 24 h at 37 °C with continuous shaking at 150 rpm to ensure uniform contact between the bacterial suspension and the sample surfaces. After incubation, serial dilutions were prepared from the suspensions, and aliquots were spread onto Mueller-Hinton agar plates. The plates were incubated at 37 °C for 24 h, and the number of colony-forming units (CFU) was counted. The antibacterial activity was calculated based on the reduction in viable bacterial count using the following formula (Equation (3)) [39]:

(3) $$Log\;Reduction=\log_{10}\left(\frac{A}{B}\right)$$

where *A* is the bacterial count at zero (initial control), and *B* is the bacterial count after 24 h of contact with the specimen. All tests were performed in triplicate (*n* = 3), and the average log reduction values were reported. According to standard interpretation criteria, specimens with at least a 1 log reduction in bacteria load are required to claim antibacterial property and greater than 2 were considered to exhibit significant antibacterial activity.

### 2.8. PVA Attachment Test

In this study, PVA (molecular weight (Mw) 125,000 g/mol, 99% hydrolyzed) was obtained from Sigma-Aldrich Co. (St Louis, MO, USA). For the preparation of PVA solution, 1 g of PVA was weighed, and 9 mL of distilled water were added to obtain solutions with a concentration of 10 wt% [40]. The prepared solutions were homogenized by mixing in a magnetic stirrer at 90 °C with a stirring speed of 500 rpm for 2 h. The casting method was employed to prepare the composite film with BC and PVA. PVA was solubilized in deionized water and stirred for 60 min at 90 °C. The PVA solution was cooled, then BC was added and agitated for 20 min. The mixture was incorporated with 30% *w*/*w* glycerol as a plasticizer. The samples were cast in a Petri dish and dried for 48 h at an ambient temperature. Washing procedures are crucial for removing excess PVA and assessing attachment efficiency, which in turn affects the composite’s mechanical and hydrophilic properties. This typically involves immersing the composites in deionized water at room temperature for a specified duration. During this time, the water-soluble PVA not chemically bonded to BC can diffuse out, which is essential for assessing the effectiveness of the incorporation process. Since the initial weights of the PVA and BC used were known, after washing, the amount of PVA remaining on the BC was calculated as a result of the final weighing (Equation (4)). The experiments were repeated three times for each initial pH value.

(4) $$PVA\;Attachment\left(\%\right)=\frac{Initial\;weight\;of\;composite-Final\;weight\;of\;composite}{Initial\;weight\;of\;composite}\times 100$$

### 2.9. Scanning Electron Microscopy (SEM)

SEM analysis was performed using a Thermo Scientific Helios 5 PFIB CXe (ThermoFisher Scientific, Waltham, MA, USA) device at 3 KeV/25 pA impact energy and 4 mm WD with an SE detector (TLD mode). Energy Dispersive Spectroscopy (EDS) analyses were performed with equipment outfitted with a Bruker XFlash 5030 detector (XFlash 5030, Bruker Co., Billerica, MA, USA). Carbon tape substrates are used to fix BC samples on the holder, subsequently gold-coated with a thickness of 30 nm.

### 2.10. Fourier Transform Infrared Spectroscopy Analysis (FT-IR)

FTIR spectroscopy was used to analyze the chemical composition, and functional groups present in the BC membrane. A Nicolet IN10 microFTIR device (Thermo Fisher Scientific, Waltham, MA, USA) operating in transmittance mode is used by measuring the absorption of infrared light with a wave number between 4,000 and 500 cm^−1^. BC4, BC6, BC7, and BC9 samples have been analyzed.

### 2.11. Statistical Analysis

The impact of initial pH on BC thickness, conductivity, tensile characteristics, and water absorption data was assessed statistically using one-way ANOVA. Prior to using ANOVA, the assumptions of normality and homogeneity of variance were confirmed. Tukey’s HSD post hoc test was used to further analyze significant effects and identify pairwise differences between pH groups. To calculate the mean ± standard deviation (SD), five replicates were used in thickness measurements for each group, and three replicates were used for other measurements. Differences were considered statistically significant at *p* < 0.05.

## 3. Results

### 3.1. Physical and Electrical Properties

#### 3.1.1. Thickness, Electrical Conductivity, and Water Absorption Capacity

**Figure 2:**
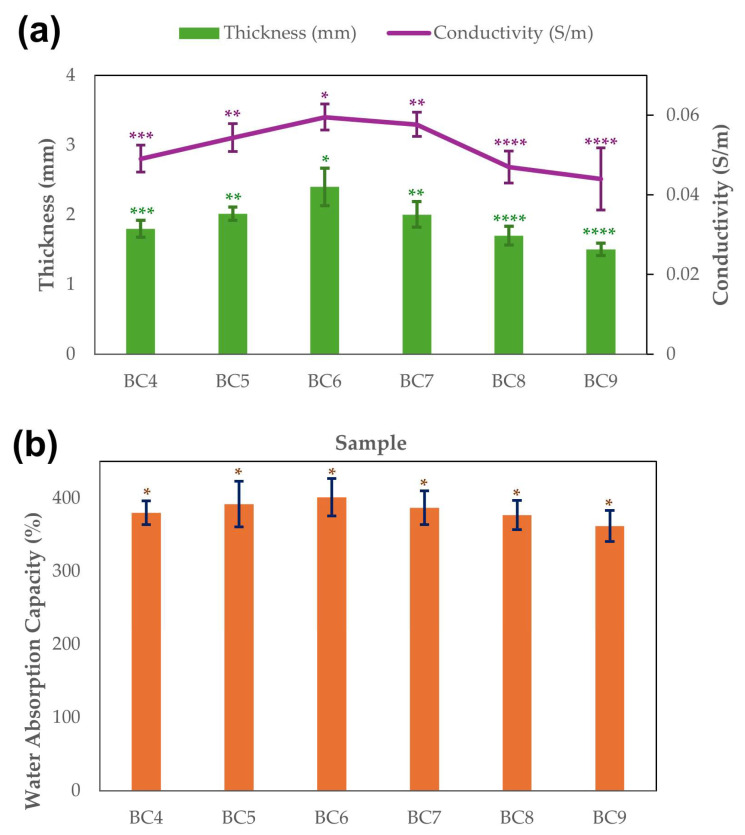
BC thickness and conductivity variations (**a**) and water absorption measurements (**b**) depending on the initial pH value. Data are presented as mean values and error bars represent the standard deviation (*n* = 5 for thickness and *n* = 3 for conductivity and water adsorption). The number of asterisks on the bars indicates statistically significant differences using one-way ANOVA followed by Tukey’s HSD post hoc test (*p* < 0.05).

Figure 2a shows the BC thickness and electrical conductivity obtained according to the initial pH value graphically. The mean thickness values ranged between 1.5 mm at pH 9 and 2.4 mm at pH 6. From pH 7, the thickness started to decrease and reached its lowest value at pH 9. This may indicate that neutral or slightly acidic pH is more suitable for optimum thickness under BC synthesis conditions. These findings indicate that pH is an important factor in BC production and that optimum pH levels affect cellulose thickness and yield.

One-way ANOVA confirmed that the initial pH had a significant effect on BC thickness (F (5,24) = 18.55, *p* < 0.0001). Tukey’s HSD post hoc test further revealed that BC synthesized at pH 6 exhibited significantly greater thickness compared to pH 4, 5, 7, 8, and 9 (*p* < 0.05). In addition, BC produced at pH 5 was significantly thicker than those at pH 8 and 9, while pH 7 samples were significantly thicker than those at pH 9. No significant differences were observed among the remaining comparisons.

In the study of BC production from waste figs using *K. xylinus* bacteria, it was revealed that *K. xylinus* produced significant amounts of BC over a wide pH spectrum (4.0–6.5) and the highest BC concentration was observed at pH 6.05 [41]. Using a new kombucha consortium (*K. saccharivorans*, *B. bruxellensis*, and *B. anomalus*) with black tea, a low-cost source, it was reported that BC could be produced at a high rate at a pH of 6 and 30 °C. The results showed that BC production increased 4.06-fold in the optimized medium compared to Hestrin-Schramm medium and the cost was reduced by 29.7% [42].

The maximum BC thickness values obtained at pH 6 (2.4 mm) are consistent with the initial pH and temperature values reported in previous studies as the parameters yielding the highest BC. However, most reports present BC yield in terms of dry weight (g/L) rather than thickness. Therefore, Table 1 provides a summary of representative studies that include both thickness and/or yield values to correlate our findings with the literature. 

The electrical conductivity of the BC samples was found to be significantly influenced by the initial pH of the medium during synthesis. As shown in Figure 2a, the conductivity values exhibited a rising trend from pH 4 to pH 6, followed by a gradual decline as the pH increased beyond this point. Specifically, the BC6 sample (pH 6) demonstrated the highest electrical conductivity value of 0.060 S/m, indicating optimal ionic mobility at near-neutral conditions. In contrast, the lowest conductivity was observed for BC9 (pH 9) at 0.044 S/m. Furthermore, also statistically, the initial pH had a substantial impact on the electrical conductivity of BC samples (F (5,12) = 10.8, *p* < 0.001). At pH 9, the mean conductivity was 0.044 S/m, while at pH 6, it was 0.060 S/m. The BC6 group (pH 6) exhibited substantially higher conductivity than pH 4, 8, and 9, according to Tukey’s HSD post hoc test. In addition, compared to pH 9, the conductivity of the pH 5 and pH 7 samples was noticeably higher. The other comparisons showed no discernible changes. According to these findings, the BC matrix’s ideal ionic mobility and conductive routes are supported by intermediate pH values (5–7), especially pH 6.

The BC samples in this study demonstrated significantly higher electrical conductivity than both unmodified plant-based cellulose and previously reported bacterial cellulose. While bamboo-derived cellulose showed only 4.0 × 10^−9^ S/cm [44] and *Acetobacter xylinum*-derived BC as low as 1.8 × 10^−11^ S/m [45], our unmodified BC samples achieved values up to 0.060 S/m, indicating a substantial improvement without chemical modification. This comparison highlights the superior intrinsic conductivity of BC used in our study, even without the integration of conductive polymers. The enhanced conductivity at pH 6 could be attributed to the optimized structural configuration and ionic mobility within the BC network. In contrast, the polypyrole-based enhancement in the referenced study required significant chemical modification and incorporation of external conductive components to achieve similar conductivity levels. Furthermore, our BC samples maintained their structural integrity and eco-friendly profile, making them particularly attractive for applications in biodegradable electronics, wearable sensors, and moisture-stable devices, without the need for synthetic additives.

A positive correlation was also noted between sample thickness and conductivity. The BC6 sample, which exhibited the maximum thickness (2.4 mm), corresponded with the highest conductivity, while the thinnest sample, BC9 (1.5 mm), showed the lowest conductivity. This finding implies that increased thickness may reflect a denser and more interconnected structure capable of facilitating better electron or ion transport pathways within the BC matrix. Taken together, these results indicate that pH 6 represents a critical condition for maximizing the conductive performance of BC-based materials, offering insights into the optimization of such biopolymers for applications requiring moderate electrical activity, such as flexible electronics, biosensors, or electroactive wound dressings.

Water retention capacity and water release rate are the most important properties directly involved in biomedical applications of BC as a dressing material and are due to the highly porous nature of BC [46]. Water is present inside the pores and is bound to cellulose fibrils through hydrogen bonding. The pH value is one of the main factors affecting this property. Approximately pH 6–6.5 is recommended in some studies for optimal properties [43,46]. Figure 2b presents the results of water absorption measurements with initial pH values ranging from 4–9 and then purifying the harvested BC membranes. The water absorption capacity of the obtained BCs increased from pH 4 to 6, reaching a maximum value of 401%. From pH 7, the absorption capacity started to decrease and reached its lowest at pH 9 with 362%. A one-way ANOVA revealed that the initial pH had no statistically significant effect on water absorption capacity (F (5,12) = 1.07, *p* = 0.425). Tukey’s post hoc analysis revealed no significant pairwise differences between pH groups (*p* > 0.05). These results suggest that water absorption capacity remains widely unaffected by pH variations, whereas conductivity and thickness exhibit greater sensitivity to changes in initial pH.

The analysis of BC thickness and electrical conductivity as a function of initial pH revealed a strong interdependence between structural and functional properties. Both two parameters showed a similar trend: increasing from pH 4 to 6, peaking at pH 6, and gradually declining beyond this point. The BC6 sample, produced at pH 6, exhibited the greatest thickness (2.4 mm), highest conductivity (0.060 S/m), and maximum water absorption capacity (401%). This indicates that near-neutral pH conditions provide optimal synthesis conditions for BC, promoting denser network formation, improved ionic mobility, and enhanced porosity. The positive correlation observed between thickness and conductivity suggests that a more interconnected cellulose matrix supports more efficient transport. Although the water absorption capacity is not significantly influenced by pH changes, increased porosity at pH 6 enhances water retention through hydrogen bonds within the cellulose network, which is particularly beneficial for biomedical applications such as wound dressing. These findings are consistent with literature reports that emphasize pH 6–6.5 as an ideal range for maximizing both structural integrity and performance characteristics of bacterial cellulose [43,46]. Overall, the study demonstrates that initial pH is a critical parameter influencing multiple functional attributes of BC and optimizing it can enhance the material’s suitability for eco-friendly electronic and biomedical applications.

#### 3.1.2. Results of Colorfastness Tests

The effects of pH levels on perspiration, rubbing, and washing fastness are presented in Table 2. Although no dye was applied to the BC samples, standardized colorfastness tests were conducted to evaluate the material’s resistance to washing, perspiration, and mechanical friction, as a proxy for structural durability in wearable and biomedical applications. All samples exhibited a top rating of 5 for both perspiration and washing resistance, indicating no visible degradation, deformation, or surface change following exposure to artificial sweat or repeated laundering cycles.

Notably, rubbing fastness, assessed via the number of dry cycles until visible surface wear, demonstrated a clear pH dependency. The BC6 sample (pH 6) showed the highest resistance (32 ± 2 cycles), while BC9 (pH 9) showed the lowest (18 ± 2 cycles). pH had a highly significant impact on rubbing resistance, according to a one-way ANOVA (F (5,12) = 24.6, *p* < 0.0001). According to the results of Tukey’s post hoc analysis, pH 6 had significantly higher fastness than pH 4, 8, and 9, and pH 5 and pH 7 were also significantly higher than pH 8 and 9. Furthermore, rubbing fastness was significantly higher at pH 4 than pH 9. The other groups did not differ significantly from one another. These findings support the notion that the ideal pH range for increasing the rubbing durability of BC samples is the most favorable pH range (5–7). This trend suggests that BC synthesized under near-neutral pH forms a denser and more mechanically robust network, consistent with conductivity and thickness results discussed earlier.

These results are particularly promising when compared with similar fastness-related studies involving BC. For instance, Harmon (2020) noted that BC films without any finishing treatments suffered surface disruption after sweat or laundering simulations [47]. Similarly, recent work on natural textile fibers such as cotton dyed with natural extracts reported significantly lower resistance to perspiration (ratings 2–3) [48]. Taken together, these findings support the potential of unmodified BC membranes not only as flexible and conductive materials but also as mechanically and chemically resilient substrates suitable for sweat-exposed and washable electronic or biomedical systems.

### 3.2. Mechanical Properties

**Figure 3:**
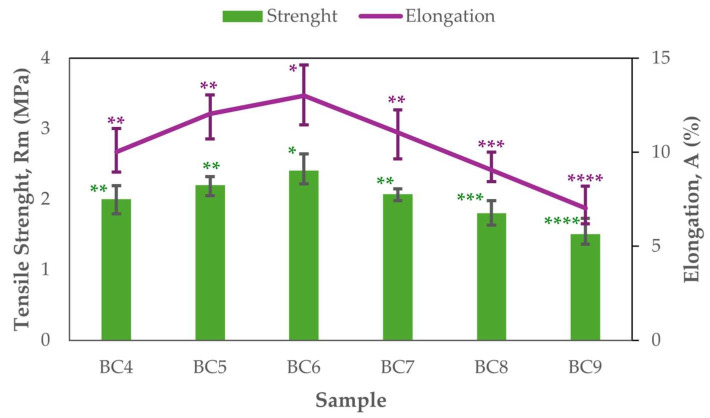
Tensile strength and elongation as a function of initial pH. Data are presented as mean values, and error bars indicate the minimum and maximum values obtained from replicate measurements (*n* = 3). The number of asterisks on the bars indicates statistically significant differences using one-way ANOVA followed by Tukey’s HSD post hoc test (*p* < 0.05).

The mechanical properties of the BC membranes demonstrated a clear dependence on the initial pH of the culture medium. As shown in Figure 3, both properties showed a similar trend, increasing from pH 4.0 and reaching maximum values at pH 6.0, followed by a gradual decline at higher pH values. The BC6 sample (pH 6.0) exhibited the highest tensile strength (2.4 ± 0.2 MPa) and elongation (13 ± 1.6%), indicating optimal nanofiber formation and cohesive network structure under near-neutral conditions. In contrast, the samples produced at pH 9.0 showed significantly lower mechanical performance, with a tensile strength of 1.5 ± 0.2 MPa and elongation of 7 ± 1.04 (%), likely due to disrupted fibrillar assembly and reduced hydrogen bonding at more alkaline conditions.

Tensile strength (F (5,12) = 9.88, *p* = 0.0006) and elongation (F (5,12) = 9.44, *p* = 0.0008) were both significantly impacted by pH, according to a one-way ANOVA. The BC6 group (pH 6) demonstrated were also significantly higher tensile strength than pH 8 and pH 9, while pH 5 and pH 7 were likewise significantly stronger than pH 9, according to Tukey’s HSD test. Elongation was also substantially higher at pH 6 than at pH 8 and 9, with pH 5 and pH 7 similarly exceeding pH 9 in this regard. These findings demonstrate that near-neutral pH, especially pH 6, provides optimal conditions for enhancing BC samples’ mechanical performance. These findings confirm that the structural integrity of BC can be fine-tuned by adjusting the pH during synthesis.

While the strength values obtained in this study (ranging from 1.5–2.4 MPa) are lower than those reported for highly purified or treated BC membranes (often >30 MPa) [16,49], they are considered suitable for applications such as soft bioelectronics, moisture-sensitive devices, or wound dressing supports. Similarly, the elongation values achieved (up to 13%) fall within or slightly below typical ranges reported for unmodified BC but demonstrate meaningful flexibility for use in deformable systems.

The BC membranes in this study were produced using *K. xylinus* ATCC 53524, a well-known high-performing strain in terms of both yield and structural quality. These results are highly consistent with those reported in previous studies for the same strain. The methodology employed in this study for BC production closely follows the approach reported by Chen et al. [50], in which various *Komagataeibacter* strains were used and demonstrated that ATCC 53,524 produced BC with superior mechanical properties, including a breaking stress of 0.68 MPa, breaking strain of 20.7%, and Young’s modulus of 5.56 MPa, under controlled conditions. However, a key difference in our protocol was the supplementation of the growth medium with tea waste, a low-cost, nutrient-rich organic byproduct. This modification introduces a novel and sustainable approach to BC production while simultaneously offering additional carbon and polyphenolic sources that may influence the nanofibrillar assembly and material performance. Despite using a similar strain and culture strategy, our BC membranes exhibited significantly higher tensile strength (up to 2.4 MPa) but lower elongation at break (max. 13%) compared to Chen’s study (0.68 MPa strength, ~20.7% elongation). This variation may arise from differences in membrane composition, structural compaction, and potential interactions between tea-derived phenolic compounds and cellulose fibers, which can enhance stiffness and reduce flexibility. Previous studies have suggested that phenolic additives can induce hydrogen bonding or act as mild crosslinking agents, leading to tighter fibril networks and altered mechanical responses.

Overall, the mechanical evaluation confirms that pH 6.0 is a critical condition for enhancing the structural performance of BC, supporting its potential for flexible and skin-contact applications. The incorporation of tea waste not only enhances the sustainability profile of the production process but also allows for modulation of BC properties by biochemical tuning of the culture medium. This aligns with current trends in green biomaterial development, where agricultural waste valorization is coupled with high-performance material synthesis. Even being lower than peak literature values, the strength and elongation values obtained here are consistent, reproducible, and sufficient for many biomedical and flexible electronics applications, particularly where moderate mechanical durability, conformability, and biocompatibility are more critical than extreme mechanical strength.

### 3.3. Morphology Analysis by SEM Images

**Figure 4:**
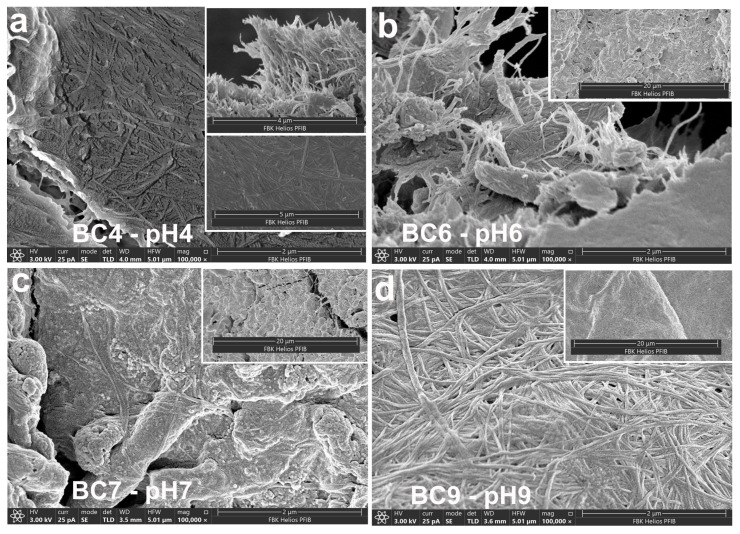
SEM images of BCs depending on the initial pH value: (**a**) pH 4, (**b**) pH 6, (**c**) pH 7, and (**d**) pH 9.

SEM images (Figure 4) demonstrate that the morphology of BC is significantly influenced by the pH of the culture medium. At pH 4 (Figure 4a) and at pH 9 (Figure 4d) presented a densely packed three-dimensional ribbon-like network of nanofibril structures. The microstructure obtained for pH 4 is consistent with the study results obtained for BC prepared with black tea and grown in Kombucha culture with initial pH 4.06–4.35 [49]. The pH 9 sample (Figure 4d) presented a densely packed but morphologically coarser network than pH 4 with visibly thicker fibers. Thicker fibers can reduce flexibility and may limit surface reactivity, which is critical for biomedical and electrode applications where intimate skin or tissue contact is required. At pH 6 (Figure 4b) and pH 7 (Figure 4c) conditions, significant coverage of the surface was obtained. This coating was observed in various areas under pH 7 conditions, accompanied by some agglomeration in the form of clumps. These clumps may have originated from the lyophilization of the sugar-rich extract [51]. The most uniform and cohesive fibrillar arrangement, on the other hand, was shown by the structure produced at pH 6 (Figure 4b), which formed a dense and well-entangled 3D nanofiber network. This shape is in line with earlier research showing that BC production is best achieved at a pH close to neutral [16,43].

**Figure 5:**
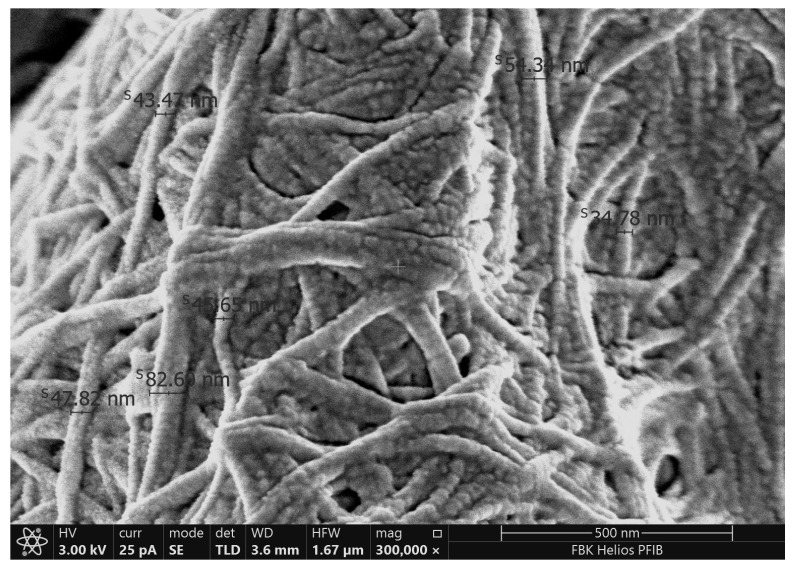
Fiber diameters measured by using SEM for BC9 sample.

Quantitative analysis of the pH 9 SEM image (Figure 5) revealed fiber diameters ranging from 24.8 nm to 82.6 nm, with an average of about 50 nm. Although this falls within the expected nanofiber scale, the increased thickness and density may limit flexibility and surface reactivity—properties critical for biomedical applications such as wound dressings and flexible electrodes. These results underscore the importance of pH as a tunable parameter for tailoring BC morphology according to specific functional requirements. These morphological observations strongly support the idea that synthesis pH can be used as a tunable design parameter for tailoring BC properties: near-neutral pH (especially pH 6) yields a tightly interwoven, fine-fibered network ideal for mechanically robust and conductive applications such as wearable electrodes, while more acidic pH (pH 4–5) produces morphologies that, despite lower mechanical cohesion, are associated with enhanced antimicrobial activity, making them better suited for infection-preventive wound dressings.

### 3.4. Fourier Transformed Infrared (FTIR) Analysis

**Figure 6:**
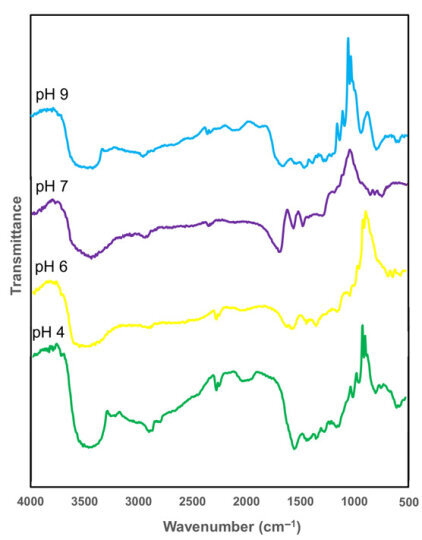
FTIR spectra of BC4, BC6, BC7, and BC9 samples.

FTIR analysis confirmed the characteristic functional groups of BCs across all samples synthesized at different pH levels (Figure 6). Broad bands around 3475–3410 cm^−1^ are attributed to O–H stretching vibrations representing the hydroxyl bonds in these cellulose samples [52]. This strong and broad O–H band also indicates an extensive hydrogen-bonding network, which is directly related to the high-water absorption capacity observed in BC membranes. Peaks near 2800–2900 cm^−1^ represent symmetric (−CH) stretching vibrations indicating the presence of methyl/methylene functional groups, consistent with typical cellulose structures [43,52]. The region around 1030–1050 cm^−1^, indicates the presence of (C–O) stretching vibration in the polymer, a feature that correlates with the intact polysaccharide backbone essential for maintaining mechanical integrity [52]. The peak at ~890 cm^−1^, corresponding to β-glycosidic linkages, further confirm the presence of amorphous cellulose absorption band [13].

Preservation of this β-linkage across all pH conditions suggests that the cellulose molecular framework remains chemically stable, even though physical and morphological characteristics vary. Although no major shifts in peak positions were detected among BC4, BC6, BC7, and BC9, slight variations in band intensity—particularly in the O–H stretching region—may reflect subtle changes in hydrogen bonding density and moisture content. For example, the more defined O–H band in BC6 aligns with its superior water absorption and tensile strength, suggesting a denser and more cohesive nanofiber network. Conversely, reduced intensity in alkaline conditions (BC9) may indicate less compact hydrogen-bonding arrangements, consistent with its lower mechanical and antibacterial performance.

This finding aligns with prior studies suggesting that near-neutral pH conditions promote optimal bacterial cellulose assembly and structural order [16,43]. Such structural preservation is essential for ensuring reliable performance in biomedical devices, where both chemical stability and physical cohesion are critical—for instance, in long-term wound dressings, skin-contact electrodes, and flexible biosensors.

### 3.5. Antimicrobial Test Results

Table 3 shows the antibacterial activity of BC specimens prepared under varying initial pH conditions, evaluated against the Gram-negative strain *E. coli*. The test was conducted using the dynamic contact method described in ASTM E2149-13a, and the results are expressed in terms of log reduction in viable bacterial count after 24 h of incubation.

All BC samples demonstrated varying degrees of antibacterial activity, with a clear correlation between initial pH conditions and antimicrobial performance. The BC4 sample, prepared under the most acidic condition, exhibited the highest antibacterial activity with a log reduction of 3.5, which corresponds to a “high” level of efficacy according to standard classification. Similarly, BC5 and BC6 displayed log reductions of 3.2 and 2.8, respectively, indicating moderate to high antibacterial effectiveness.

As the initial pH increased toward neutral and alkaline conditions, the antibacterial activity progressively declined. BC7 and BC8 samples showed log reductions of 2.5 and 2.0, respectively, reflecting low to moderate activity. The BC9 specimen, associated with the most alkaline condition, exhibited the lowest antibacterial activity with a log reduction of 1.5, which falls below the threshold of significant antimicrobial effectiveness.

*E. coli* is reported to grow well under weakly acidic to neutral conditions but is inhibited at low pH [53]. These study’s findings suggest that acidic pH conditions during BC preparation enhance the antibacterial potential of the material. This effect may be attributed to changes in surface chemistry, such as increased negative charge density, porosity, or enhanced interaction between bacterial membranes and the BC matrix. The results further reinforce the notion that modulating the pH during synthesis can be an effective strategy to tune the antimicrobial properties of bacterial cellulose-based materials without incorporating external biocidal agents. Although *E. coli* is known for acid resistance the antimicrobial effect might be triggered by the changes in surface due to pH.

### 3.6. PVA Attachment Test Results

Polyvinyl alcohol (PVA) is a widely used polymer in film production, textile, medicine, and agriculture due to its water solubility and non-toxic biodegradability. However, PVA has incomplete mechanical properties, often requiring costly additives like starch and sodium alginate to enhance its performance. Studies have shown that incorporating cellulose improves PVA’s properties, but plant-derived cellulose requires complex purification processes [54,55]. PVA and BC form a composite material with enhanced mechanical strength, hydrophilicity, and structural integrity. The interaction between hydroxyl groups in both materials allow for tailored water absorption, making the composite useful in various applications, including food packaging and biomedical engineering [54,55].

Table 4 presents the PVA attachment percentages for BC–PVA composite samples prepared under varying pH conditions. The results were calculated based on weight loss after the washing step, which removes non-bonded, water-soluble PVA. This parameter provides insight into the effectiveness of PVA incorporation into the BC matrix and its potential contribution to the final material properties.

The results indicate that PVA attachment values ranged between 5% and 12%, with the highest incorporation observed in BC6 (12%), followed by BC5 and BC7 (10%). On the other hand, BC9 exhibited the lowest attachment efficiency (5%), which may be due to structural changes in the BC network at high pH conditions that limit hydrogen bonding between PVA and BC. The impact of pH was shown to be statistically significant by one-way ANOVA (F (5,12) = 11.28, *p* = 0.0003). The results of Tukey’s test showed that samples at pH 6 had significantly higher attachment than samples at pH 4, 8, and 9, and that samples at pH 5 and pH 7 had significantly higher attachment than those at pH 9 (*p* < 0.05). According to these findings, PVA adherence to BC structures is improved by moderate pH levels, particularly pH 6, thereby enhancing interfacial compatibility with polymer matrices. Since the interaction between hydroxyl groups of BC and PVA is primarily responsible for network formation, pH-induced disruption of these groups may reduce the efficiency of physical entrapment and bonding.

## 4. Conclusions

This study demonstrated the production of bacterial cellulose (BC) using *Komagataeibacter xylinus* and spent black tea waste as a low-cost and sustainable carbon source. The physicochemical, mechanical, antimicrobial, and electrical properties of the synthesized BC membranes were strongly influenced by the initial pH of the culture medium. Among the tested conditions, pH 6.0 yielded the most favorable results in terms of tensile strength (2.4 MPa), elongation (13%), water absorption, electrical conductivity, and PVA attachment (12%), indicating a highly cohesive nanofiber network. The combination of high tensile strength, water retention, and electrical conductivity obtained at pH 6 makes these BC membranes particularly suitable for flexible electrodes, biosensors, and wearable biomedical devices. Furthermore, BC membranes produced at acidic pH values (pH 4–6) exhibited higher antibacterial activity, with log reductions exceeding 2.8 against *E. coli*, likely due to enhanced surface chemistry and porosity. The strong antibacterial activity observed at pH 4–6 indicates their potential use in infection-prone biomedical applications such as wound dressings, surgical patches, and antimicrobial coatings.

FTIR and SEM analyses confirmed the preservation of key cellulose functional groups and revealed structural differences depending on pH with the most uniform nanofibrillar morphology observed at pH 6, respectively. PVA attachment tests further demonstrated that moderate pH conditions enhance composite integration. Moderate PVA incorporation in BC membranes, as observed in BC5 and BC6, offers a balance between mechanical durability and biocompatibility, which is advantageous for moist-contact biomedical materials like electrotherapy pads and biopotential measurement patches.

In addition to offering functional and performance advantages, the use of food industry waste (spent tea leaves) as a feedstock contributes to environmental sustainability by promoting circular bioeconomy principles. This approach directly supports the United Nations Sustainable Development Goals, particularly SDG 12 (Responsible Consumption and Production) through waste valorization and resource efficiency, and SDG 13 (Climate Action) by reducing carbon-intensive input materials. The valorization of spent tea waste not only addresses environmental concerns but also provides a sustainable pathway for the mass production of functional BC-based biomaterials in medical device, packaging, and wearable technology industries.

Taken together, this work presents a viable and potentially scalable process for producing BC-based materials that may be applied in biomedical devices such as flexible electrodes and antimicrobial wound dressings. Nevertheless, several challenges remain in translating the process from laboratory to industrial scale, including limited oxygen and nutrient diffusion in larger containers, the risk of contamination when utilizing agro-waste substrates, and the inherent variability in tea waste composition, which may affect batch-to-batch consistency. Economic considerations, including downstream purification and substrate preparation, must also be addressed. Despite these challenges, the findings demonstrate that tea waste is a sustainable and feasible feedstock for BC synthesis. Future research will be directed toward cost estimation, bioreactor-based scale-up, and the integration of BC composites into functional device platforms.

## Figures and Tables

**Table 1:** Summary of representative studies reporting thickness and/or yield values.

Study (Ref.)	Carbon Sources/Medium	Bacteria	Vessel Type & Size	pH	Temperature (°C)	Incubation Time	BC Thickness (mm) or Yield (g/L)	Notes
Present study	Waste black tea medium	*K. xylinus*	Erlenmeyer flasks (250 mL)	4–9	30	10 days	1.5–2.4 mm	Maximum at pH 6
[41]	Waste fig extract + 20 g/L of initial sugar	*K. xylinus*	Erlenmeyer flasks (250 mL)	4–6.5	25–35	7 days	8.45 ± 0.51 g/L	Maximum at 6.05 and 30 °C
[42]	1% black tea and 6% glucose	*K. saccharivorans*, *B. bruxellensis*, and *B. anomalus*	-	6	30	10 days	18.68 g/L	29.74% cost reduction
[16]	Kombucha consortium + HS medium	*K. rhaeticus* K23	Erlenmeyer flasks (250 mL)	4–5.5	26–32	7–14 days	0.52–9.1 g/L	The maximum BC production, 9.1 ± 0.66 g/L, was obtained from 32 °C, pH 5.5, 8 log CFU·mL^−1^ and 14 days of incubation
[43]	*V. faba* plants, 1.5% glucose and a 0.15% yeast extract	*Rhizobium* sp. and *K. hansenii*	500 mL flask	5–7	25–35	7 days	10–35 µm, 2.5 g/L	The maximum BC production, 2.5 g/L, at 30 °C and pH 6.5

**Table 2:** Fastness properties of BC depending on pH.

Specimen No.	Initial pH	Fastness		
		Perspiration	Washing	Rubbing (Cycle)
BC4	4	5	5	24 ± 3
BC5	5	5	5	28 ± 2
BC6	6	5	5	32 ± 2
BC7	7	5	5	30 ± 2
BC8	8	5	5	22 ± 2
BC9	9	5	5	18 ± 2

**Table 3:** Antimicrobial Activity (Log Reduction) of BC depending on pH.

Specimen No.	Antimicrobial Activity (Log Reduction)	Antimicrobial Activity Level
BC4	3.5	High
BC5	3.2	Moderate-High
BC6	2.8	Moderate
BC7	2.5	Low-Moderate
BC8	2.0	Low
BC9	1.5	Very Low

**Table 4:** PVA Attachment efficiency (%) of BC–PVA composites prepared under different initial pH values.

Specimen No.	PVA Attachment (%)
BC4	8 ± 0.8
BC5	10 ± 0.8
BC6	12 ± 1.4
BC7	10 ± 1.4
BC8	7 ± 0.8
BC9	5 ± 0.8

## Data Availability

The original contributions presented in this study are included in the article. Further inquiries can be directed to the corresponding author(s).

## Abbreviations

The following abbreviations are used in this manuscript:

MCC	Microcrystalline cellulose
NCC	Nanocrystalline cellulose
BC	Bacterial cellulose
AAB	Acetic acid bacteria
STLs	Spent tea leaves
ATCC	American type culture collection
PVA	Polyvinyl alcohol
